# *Eriobotrya* Belongs to *Rhaphiolepis* (Maleae, Rosaceae): Evidence From Chloroplast Genome and Nuclear Ribosomal DNA Data

**DOI:** 10.3389/fpls.2019.01731

**Published:** 2020-02-07

**Authors:** Bin-Bin Liu, Guang-Ning Liu, De-Yuan Hong, Jun Wen

**Affiliations:** ^1^ State Key Laboratory of Systematic and Evolutionary Botany, Institute of Botany, Chinese Academy of Sciences, Beijing, China; ^2^ Department of Botany, National Museum of Natural History, Smithsonian Institution, Washington, DC, United States; ^3^ College of Architecture and Urban Planning, Tongji University, Shanghai, China

**Keywords:** chloroplast genome, *Eriobotrya*, hybridization, Maleae, nrDNA, *Rhaphiolepis*, Rosaceae

## Abstract

The *Eriobotrya-Rhaphiolepis* (ER) clade consists of about 46 species distributed in East and Southeast Asia. Although *Eriobotrya* and *Rhaphiolepis* have been supported to form a clade, the monophyly of *Eriobotrya* and *Rhaphiolepis* at the genus level has never been well tested and their phylogenetic positions in Maleae still remain uncertain. This study aims to reconstruct a robust phylogeny of the ER clade in the framework of Maleae with a broad taxon sampling and clarify the phylogenetic relationship between *Eriobotrya* and *Rhaphiolepis*. This study employed sequences of the whole plastome (WP) and entire nuclear ribosomal DNA (nrDNA) repeats assembled from the genome skimming approach and included 83 samples representing 76 species in 32 genera of Rosaceae, especially Maleae. The Maximum Likelihood (ML) and Bayesian Analysis (BI) based on three datasets, i.e., WP, coding sequences of plastome (CDS), and nrDNA, strongly supported the paraphyly of *Eriobotrya*, within which *Rhaphiolepis* was nested. Our plastid tree supported the sister relationship between the ER clade and *Heteromeles*, and the nrDNA tree, however, did not resolve the phylogenetic placement of the ER clade in Maleae. Strong incongruence between the plastid and the nuclear trees is most likely explained by hybridization events, which may have played an important role in the evolutionary history of the ER clade. Molecular, morphological, and geographic evidence all supports the merge of *Eriobotrya* with *Rhaphiolepis*, which has the nomenclatural priority. We herein transferred 36 taxa of *Eriobotrya* to *Rhaphiolepis*. We also proposed a new name, *Rhaphiolepis loquata* B.B.Liu & J.Wen, for the economically important loquat, as the specific epithet “japonica” was pre-occupied in *Rhaphiolepis*.

## Introduction

Rosaceae is a widely distributed and economically important plant family currently classified into three subfamilies: Rosoideae, Dryadoideae, and Amygdaloideae (Morgan et al., 1994; Potter et al., 2007). The apple tribe Maleae consists of more than 1,000 species, mainly distributed in the temperate regions of the Northern Hemisphere with several genera extending to subtropical and tropical Southeast Asia and South America (Phipps et al., 1990; Lo and Donoghue, 2012). The apple tribe includes a number of commercially important fruits, such as apples (*Malus domestica* (Suckow) Borkh.), pears (*Pyrus* L. spp.), loquats (*Eriobotrya japonica* (Thunb.) Lindl.), as well as some ornamentals, e.g. serviceberries (*Amelanchier* Medik. spp.), chokeberries (*Aronia* Medik. spp.), and photinias (*Photinia* Lindl. spp.). Due to the economic significance, Maleae has intrigued taxonomists, botanists, horticulturists, and agriculturists for several hundred years. Furthermore, the phylogenetic position of the *Eriobotrya* Lindl.-*Rhaphiolepis* Lindl. complex within Maleae has been uncertain, perhaps due to the low sequence divergence, hybridizations, and the extensive extinctions of its close relatives (Campbell et al., 2007; Liu et al., 2019). The phylogenetic relationships among the members of Maleae have never been resolved confidently using either the morphological characteristics (Phipps et al., 1991; Aldasoro et al., 2005) or the limited plastid and/or nuclear sequences (Campbell et al., 1995; Verbylaitė et al., 2006; Campbell et al., 2007; Li et al., 2012; Lo and Donoghue, 2012; Sun et al., 2018).

The generic delimitation in Maleae has been notoriously difficult, which may be due to the low sequence divergence resulted from ancient, rapid radiations (Wolfe and Wehr, 1988; Campbell et al., 2007) and/or frequent hybridizations (Robertson et al., 1991; Lo and Donoghue, 2012; Liu et al., 2019). As the one of the few genera of Maleae largely distributed in subtropical and tropical regions (Robertson et al., 1991), *Eriobotrya* consists of ca. 15–20 species ranging from the Himalayas throughout continental southeast Asia to Japan and the islands of western Malesia (Kalkman, 2004). The well-known fruit loquat, *Eriobotrya japonica*, has been widely cultivated all over the world, making *Eriobotrya* of great economical importance. Characterized by persistent sepals and craspedodromous lateral veins of leaves, *Eriobotrya* has been treated to be distinct from *Rhaphiolepis* in all taxonomic literature and floras (Vidal, 1968; Vidal, 1970; Robertson et al., 1991; Kalkman, 1993; Lu et al., 2003; Kalkman, 2004). The monophyly of the *Eriobotrya-Rhaphiolepis* complex has been strongly supported by recent molecular phylogenetic studies based on limited taxon sampling, however, its phylogenetic placement was still controversial (Campbell et al., 2007; Li et al., 2009; Li et al., 2012; Lo and Donoghue, 2012; Liu et al., 2019; Xiang et al., 2017; Yang et al., 2012; Zhang et al., 2017). In addition, with the synapomorphies of the proportionally large seed with rounded or wide-elliptic cross-section and the lack of endosperm (Aldasoro et al., 2005; Rohrer et al., 1991), *Eriobotrya* and *Rhaphiolepis* were morphologically distinct from other members of Maleae. Although the nuclear *Adh* topology indicated the nonmonophyly of *Eriobotrya* with *Rhaphiolepis* nested within it, Yang et al. (2012) did not provide any discussions on the taxonomic implications.

As a rapid and cost-effective method in phylogenomics, genome skimming (i.e. low-coverage genome shotgun sequencing) could generate a large number of phylogenetically informative sites from the whole chloroplast genome, partial mitochondrial genome, and entire nuclear ribosomal DNA (nrDNA) repeats (Straub et al., 2012; Zhang et al, 2015; Zhang et al, 2017; Zimmer and Wen, 2015). Strongly supported incongruence between the maternally inherited plastomes and nuclear markers has been utilized to address polyploidy and reticulate evolution (Bock et al., 2014; McKain et al., 2018; Liu et al., 2019). Recently Liu et al. (2019) have successfully clarified the phylogenetic relationships between *Photinia* and its morphologically similar allies in the framework of Maleae using whole chloroplast genomes and nuclear ribosomal DNA (nrDNA) repeats assembled from genome skimming approach. The case study showed great potential in resolving the phylogenetic relationships in Maleae using genome skimming data. These datasets have been successfully applied to reconstruct the phylogenetic history of various plant lineages (e.g., in Bock et al., 2014; Zhang et al., 2015; Dillenberger et al., 2018; Mabry and Simpson, 2018; Thomson et al, 2018; Wen et al., 2018; Liu et al., 2019).

The main objectives of the present study were to clarify the phylogenetic placements of *Eriobotrya* and *Rhaphiolepis* with a broader sampling using sequences of the whole chloroplast genomes and entire nrDNA repeats via genome skimming. We aim to (1) explore the phylogenetic position of the *Eriobotrya-Rhaphiolepis* complex; (2) test the monophyly of *Eriobotrya* and clarify its phylogenetic relationship with *Rhaphiolepis*; and (3) discuss the taxonomic implications of the new phylogenetic results.

## Materials and Methods

### Sampling, DNA Extraction, and Sequencing

**Figure 1:**
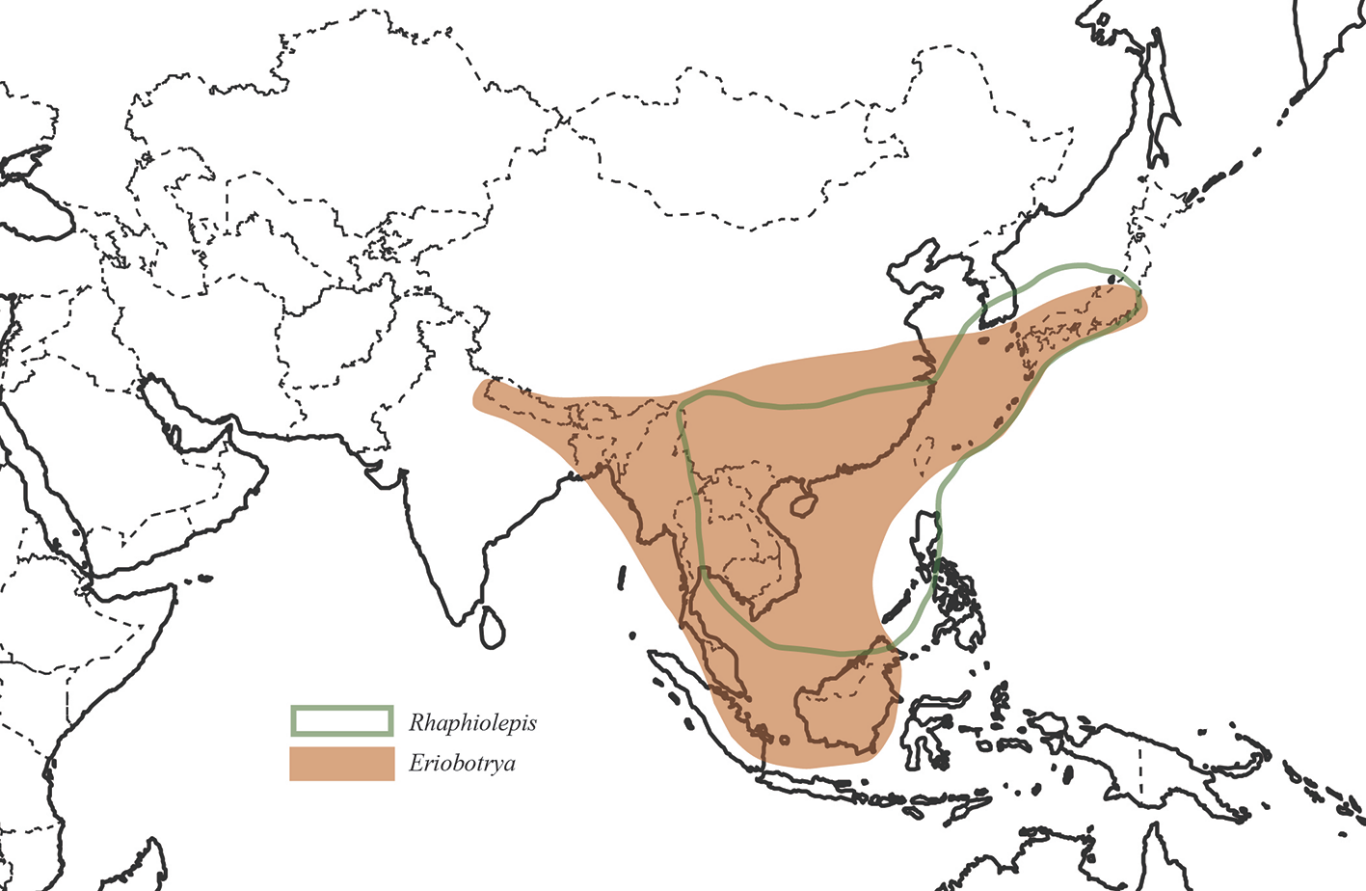
Distribution of *Eriobotrya* and *Rhaphiolepis*.

We used 83 chloroplast genomes and 68 nrDNA repeats for this analysis, in which 37 samples were sequenced for this study, 31 samples were from our previous study (Liu et al., 2019), and 15 accessions of plastomes were from GenBank. These 83 samples of plastomes represented 76 species and 32 genera which represented nearly all the genera recognized currently in Maleae expect *Chamaemeles* Lindl. As the core ingroup, nine species of *Rhaphiolepis* and 12 species of *Eriobotrya* were included in the phylogenetic analysis, representing all the morphological and geographic diversity (**Figure 1**). In order to resolve the phylogenetic positions of the *Eriobotrya*-*Rhaphiolepis* complex, we extensively sampled its closely related genera in the plastid tree, i.e., *Cotoneaster* Medik., *Heteromeles* M.Roem., and *Photinia*, as well as its closely related genus *Stranvaesia* Lindl. in the nrDNA tree (Liu et al., 2019). We also sampled 67 accessions of plastomes from 30 genera of Maleae as the ingroup, i.e., *Amelanchier*, *Aronia*, *Chaenomeles* Lindl., *Cormus* Spach, *Cotoneaster*, *Crataegus* L., *Cydonia* Mill., *Dichotomanthes* Kurz, *Docynia* Decne., *Eriolobus* (DC.) M.Roem., *Hesperomeles* Lindl., *Heteromeles*, *Kageneckia* Ruiz & Pav., *Lindleya* Kunth, *Malacomeles* (Decne.) Decne., *Malus* Mill., *Mespilus* L., *Osteomeles* Lindl., *Peraphyllum* Nutt., *Phippsiomeles* B.B.Liu & J.Wen, *Photinia*, *Pourthiaea* Decne., *Pseudocydonia* (C.K.Schneid.) C.K.Schneid., *Pyracantha* M.Roem., *Pyrus*, *Sorbus* L., *Stranvaesia*, *Torminalis* Medik., and *Vauquelinia* Corrêa ex Bonpl. The most closely related genus of Maleae, *Gillenia* was selected as the outgroup. As for the nrDNA analysis, 68 accessions representing 67 species and 29 genera were used herein.

The total genomic DNAs sequenced for this study were extracted either from silica-gel dried leaves or from the specimens in herbarium PE and US (**Supplementary Table 1**). The DNAs of the samples with species names marked with α (**Supplementary Table 1**) were extracted with a modified CTAB (mCTAB) method (Li et al., 2013b) in the lab of the State Key Laboratory of Systematic and Evolutionary Botany, CAS, China. Library preparations were done at Novogene, Beijing, China using NEBNext^®^ Ultra^™^ II DNA Library Prep Kit and the libraries were sequenced on the NovaSeq 6000 Sequencing System. The DNAs of the samples with β (**Supplementary Table 1**) were extracted using Qiagen DNeasy^®^ plant mini-kit (Qiagen Gmbh, Hilden, Germany) following the manufacturer's protocol and the libraries were prepared with the same kit in the Laboratories of Analytical Biology (LAB), National Museum of Natural History (NMNH), Smithsonian Institution (SI), USA. The pooled libraries were shipped to Novogene in UC Davis Sequencing Centre, Davis, California, USA on the Illumina HiSeq 2500 instrument. Paired-end reads of 2 × 150 bp were generated on both sequencing platforms.

### Chloroplast Genome and nrDNA Assembly, Annotation, Gene Map, and Alignment

We removed the adaptors introduced by Illumina sequencing using cutadapt 2.4 (Martin, 2011) with AGATCGGAAGAGC as the forward and the reverse adaptor. The results were checked for quality control with FastQC 0.11.8 (Andrews, 2018). NOVOPlasty 3.6 (Dierckxsens et al., 2016) was then used to assemble the plastomes *de novo* with the ribulose-1,5-bisphosphate carboxylase/oxygenase large subunit (*rbcL*) gene sequence as the seed. Twenty-two circular plastomes (59.5%) of the 37 samples were assembled with NOVOPlasty. The remaining 15 samples were assembled using Zhang et al. (2015)'s successive method combining mapping-based and *de novo* assembly. For these 15 samples, the raw data generated from the Illumina HiSeq runs were trimmed using Trimmomatic v.0.39 (Bolger et al., 2014), removing bases below PHRED 15 within a sliding window of four bases, keeping only reads of 36 bases or longer. The results were also checked by FastQC for quality control. Zhang's successive assembly used a multistep approach, including reference-guided assembly and *de novo* assembly to obtain a high-quality plastome. The reference-guided assemblies were performed by Bowtie2 2.3.5.1 (Langmead and Salzberg, 2012) using only the forward and reverse paired reads generated by Trimmomatic v.0.39 (Bolger et al., 2014). We used the closely related plastomes generated from the previous step of this study or the GenBank as the reference genome. After obtaining a complete plastome for one species, we used it as the reference to assemble its closely related samples (see Zhang et al., 2015). The resulting plastomes were used as subsequent references. *De novo* assemblies were constructed by SPAdes 3.13.1 with careful error correction and K-mer length of 21, 33, 55, 77 (Nurk et al., 2013). To correct errors and ambiguities resulting from each approach, the scaffolds obtained by *de novo* assembly, in combination with the contigs generated from NOVOPlasty were mapped to the plastome from the reference-guided assembly. Through the combined effort using NOVOPlasty and Zhang et al. (2015)'s method, we assembled high-quality plastomes for all samples.

We also used Zhang et al. (2015)'s method to assemble the entire nrDNA repeats. The raw data were trimmed by Trimmomatic v.0.39 (Bolger et al., 2014). We used the previously assembled nrDNA sequences (*Eriobotrya cavaleriei* (H.Lév.) Rehder [GenBank accession no. MN215982]and *E. deflexa* (Hemsl.) Nakai [GenBank accession no. MN215978]) to capture and map the reads to reference by Bowtie2 2.3.5.1 (Langmead and Salzberg, 2012). The scaffolds generated by SPAdes 3.13.1 (Nurk et al., 2013) in assembling the plastome were mapped to the consensus sequences, and the gaps and ambiguous sites generated by the reference method were corrected. If the quality of nrDNA sequences from *de novo* assembly was not good, we used a different reference to assemble the sequences again with the above procedure. Generally, we obtained high-quality nrDNA sequences using this successive approach that combined the strengths of reference-guided and *de novo* approaches.

The assembled plastid genomes and nrDNA repeats were annotated using Geneious Prime (Kearse et al., 2012) with a closely related and well-annotated sequence downloaded from NCBI as a reference (KT633951), and the results of automated annotation were checked manually. The coding sequences of plastome were translated into proteins for checking the start and stop codons manually in Geneious Prime. With the two reverse complementary repeats in the plastome of Rosaceae, the boundary of the large-single copy (LSC), small-single copy (SSC), and inverted-repeats (IRs) for each plastome were verified by the Find Repeats in Geneious Prime. Eighty-three plastomes were aligned by MAFFT v.7.409 (Nakamura et al., 2018) with default parameters. To reduce the systematic errors produced by poor alignment, we used the trimAL v.1.2 (Capella-Gutiérrez et al., 2009) with a heuristic method to decide on the best-automated method to trim the alignment of the whole plastome (WP). All 79 coding sequences (CDSs) of each plastome were extracted separately by Geneious Prime (Kearse et al., 2012), each coding sequence was aligned by MAFFT as specified above, and the alignment of each gene was concatenated by AMAS (Borowiec, 2016). Sixty-eighty nrDNA sequences were also aligned by MAFFT as specified above, and each region of nrDNA was extracted separately by Geneious Prime (Kearse et al., 2012), and then concatenated by AMAS (Borowiec, 2016). The gene map of *Eriobotrya japonica* and *Rhaphiolepis indica* (L.) Lindl. ex Ker Gawl. were generated by OrganellarGenomeDRAW (OGDRAW) version 1.3.1 (Greiner et al., 2019).

### Phylogenetic Analyses

We first performed independent phylogenetic analyses for each dataset (WP and nrDNA) obtained *via* genome skimming using the Maximum Likelihood (ML) and Bayesian Inference (BI). As the sequence of two IR copies was completely or nearly identical, only one copy of inverted repeat (IR) region was included for the whole plastome (WP) analyses. We used all the 79 coding sequences (CDSs) of each plastome extracted separately by Geneious Prime for phylogenetic analyses. For nrDNA sequences, the intergenic spacer (IGS) and external transcribed spacer (ETS) regions were difficult to align, only part of ETS and the complete sequences of 18S, ITS1, 5.8S, ITS2, and 26S in nrDNA were used for the phylogenetic analyses.

The best-fit partitioning schemes and nucleotide substitution models for the coding sequences of whole plastome and nrDNA dataset were estimated using PartitionFinder2 (Stamatakis, 2006; Lanfear et al., 2016) for unpartitioned whole plastome or partitioned coding sequences (CDS) of plastomes and nrDNA sequences. Under the corrected Akaike information criterion (AICc) and linked branch lengths, the PartitionFinder2 were performed by greedy (Lanfear et al., 2012) and rcluster (Lanfear et al., 2014) algorithm option for WP, plastid CDS, and nrDNA datasets, respectively, with prior defined data blocks by codon positions of each protein-coding genes and all models. The partitioning schemes and evolutionary model for each subset were used for the downstream ML and BI analyses. The ML tree was inferred by IQ-TREE v.1.6.9 (Nguyen et al., 2015) with 1000 bootstrap replicates using UFBoot2 (Hoang et al., 2017) and collapsing near zero branches option. The BI was performed with MrBayes 3.2.7 (Ronquist et al., 2012). The Markov Chain Monte Carlo (MCMC) analyses were run for 10,000,000 generations. The stationarity was regarded to be reached when the average standard deviation of split frequencies remained below 0.01. Trees were sampled at every 1,000 generations, and the first 25% of samples were discarded as burn-in. The remaining trees were used to build a 50% majority-rule consensus tree. The ML and BI trees were visualized using Geneious Prime (Kearse et al., 2012).

## Results

**Figure 2:**
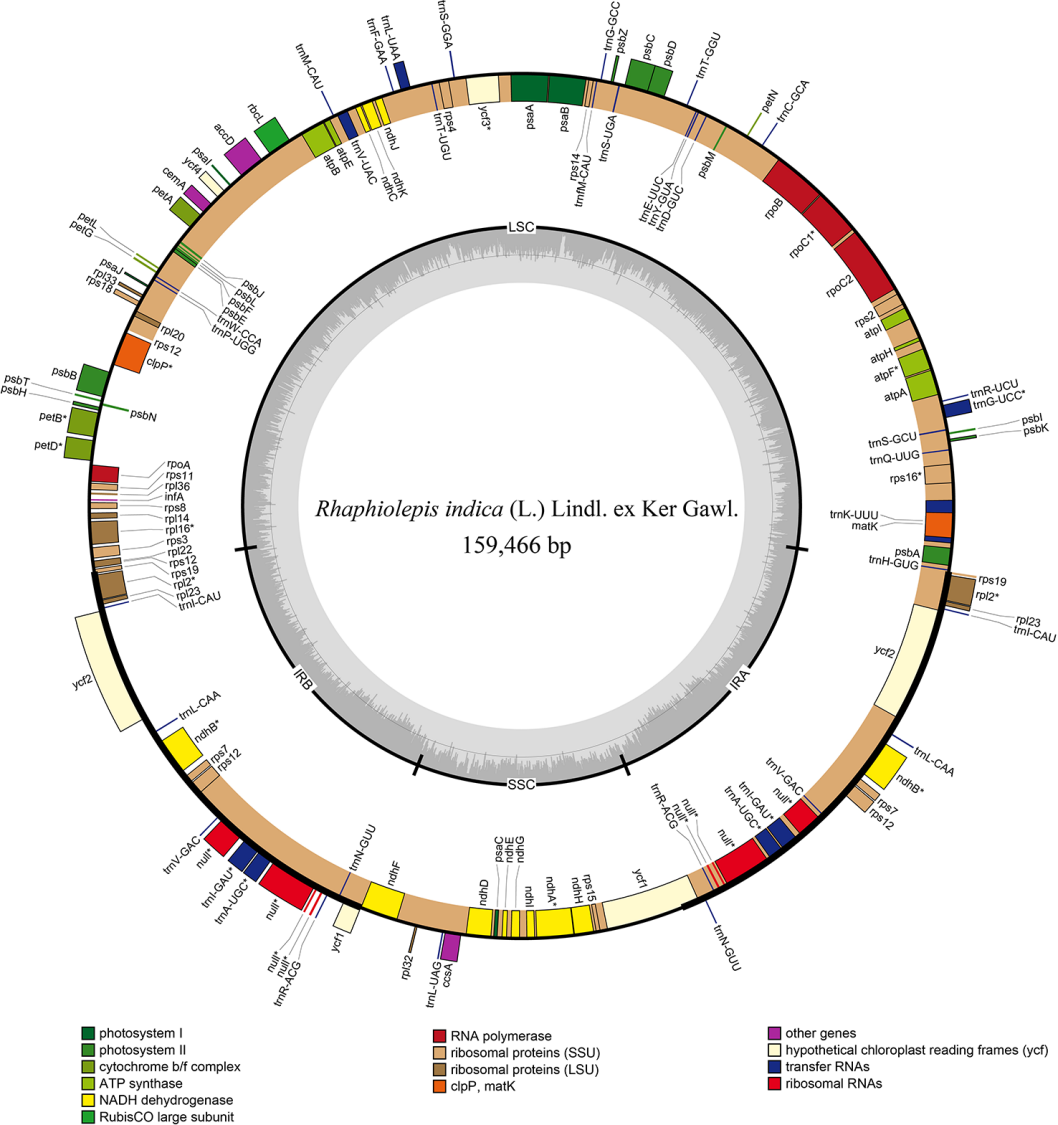
Gene map of the chloroplast genome of *Rhaphiolepis indica* (L.) Lindl. ex Ker Gawl. The genes inside and outside of the circle are transcribed in the clockwise and counterclockwise directions, respectively. Genes belong to the different functional group are shown in different colors. The thick lines indicate the extent of the inverted repeats (IRa and IRb) that separate the genomes into small single-copy (SSC) and large single-copy (LSC) regions.

The chloroplast genome of *Rhaphiolepis indica* (the type species of *Rhaphiolepis*) was 159,466 bp in length with a classic quadripartite structure that comprised of inverted repeat's pairs (**Figure 2**), and that of *Eriobotrya japonica* (type species of *Eriobotrya*) had the same structure as *R. indica* with a length of 159,156 bp (**Supplementary Figure 1**). They contained the same number of coding sequences (79), tRNAs (37), and rRNAs (8). No significant rearrangements or gene losses were found in the other species of *Eriobotrya* and *Rhaphiolepis*.

**Figure 3:**
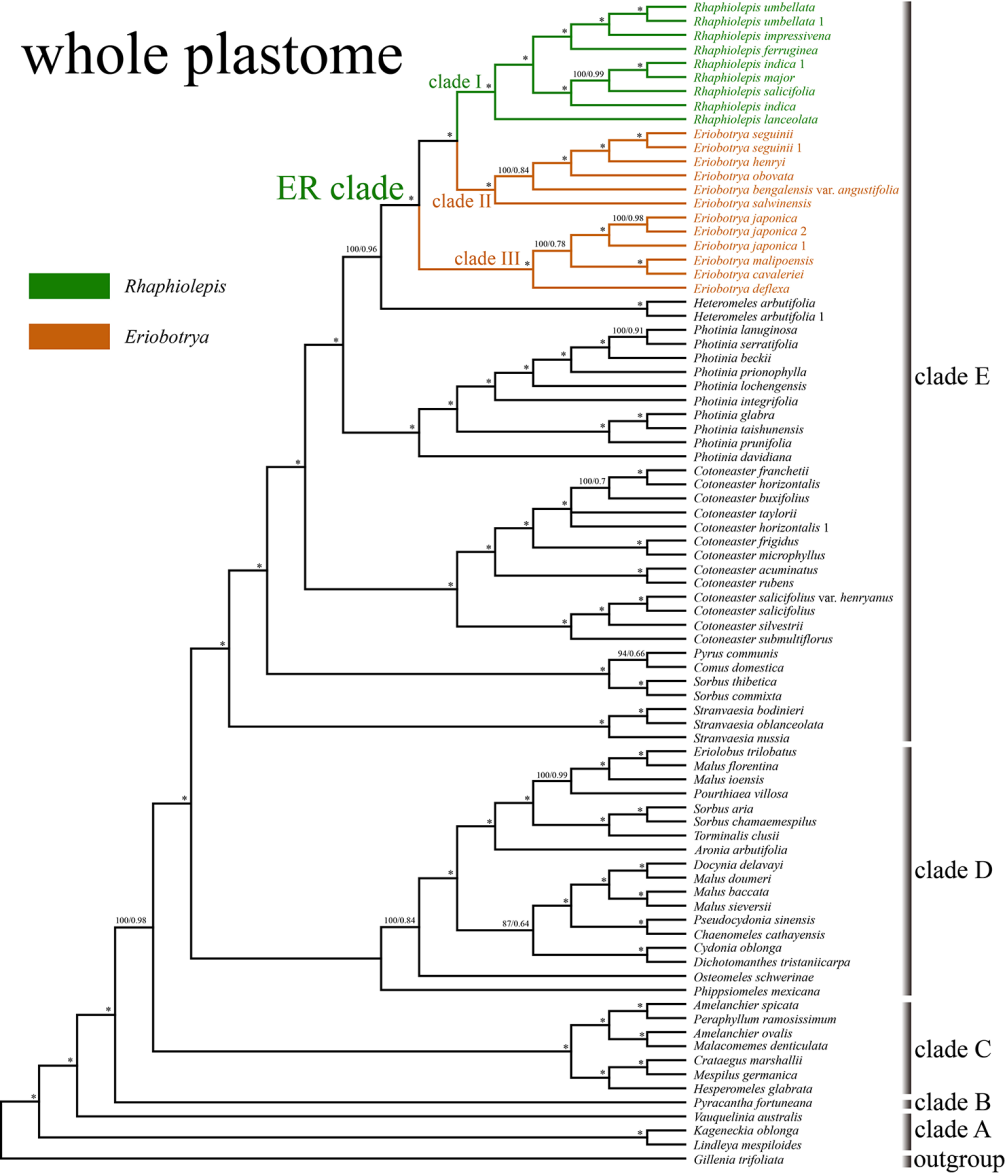
The phylogenetic relationships between *Eriobotrya* and *Rhaphiolepis* in the framework of Maleae resolved by Bayesian inference of the whole plastid genome (WP). Numbers associated with the branches are ML bootstrap value (BS) and BI posterior probabilities (PP), and asterisks (*) indicate bootstrap support/posterior probability of 100/1.00.

The aligned matrix of the 83 whole chloroplast genomes was 129,168 bp in length with the pool alignment trimmed by trimAL (Capella-Gutiérrez et al., 2009). The best-fit model of nucleotide substitutions for the ML analysis was TVM+I+G calculated by PartitionFinder, and that for the BI analysis was GTR+Γ+I. The 79 concatenated CDS sequences from 83 plastomes were generated an aligned matrix of 67,961 bp in length, which was split into 25 sets of sites (aka data blocks) with the same best scheme for each data block for the subsequent ML and BI analysis. The ML and BI analysis of WP dataset resulted in five strongly supported clades within Maleae (**Figure 3**): clade A (*Kageneckia* and *Lindleya*), clade B (only *Vauquelinia*), clade C (only *Pyracantha*), clade D (*Amelanchier*, *Crataegus*, *Hesperomeles*, *Malacomeles*, and *Peraphyllum*), and two large clades (E and F). These five major clades were also recovered by the phylogenetic analysis of CDS (**Supplementary Figure 2**). Two tropical American genera (*Kageneckia* and *Lindleya*) formed clade A, which constituted the first diverged major clade of Maleae. Clade A was sister to the North American genus *Vauquelinia* (clade B), and then together they were sister to the core Maleae (**Figure 3** and **Supplementary Figure 2**). As the basalmost group of the core Maleae, *Pyracantha* (clade C) was sister to a large clade, including clades D, E, and F. Most of the members of clade D were from the New World, and those of clades E and F were from Eurasia except that *Aronia* and *Heteromeles* are from North America. The two major Eurasian clades (E and F) were sister to each other, and then together sister to the New World clade D (**Figure 3** and **Supplementary Figure 2**).

Samples of *Eriobotrya* and *Rhaphiolepis* were strongly supported to form a monophyletic group (BS = 100, PP = 1) by the WP and CDS trees (**Figure 3** and **Supplementary Figure 2**). Although *Rhaphiolepis* was recovered as a monophyletic group (clade I) with strong support (WP: BS = 99, PP = 1; CDS: BS = 96, PP = 1), *Eriobotrya* was divided into two clades, clades II & III, with the *Rhaphiolepis* clade (I) sister to clade II (*E. henryi* Nakai, *E. obovata* W.W.Sm., *E. salwinensis* Hand.-Mazz., and *E. seguinii* (H.Lév.) Cardot ex Guillaumin), and together they were sister to clade III (*E. cavaleriei*, *E. deflexa*, *E. japonica*, and *E. malipoensis* K.C.Kuan). *Eriobotrya* was also paraphyletic in these two trees (**Figure 3** and **Supplementary Figure 2**).

**Figure 4:**
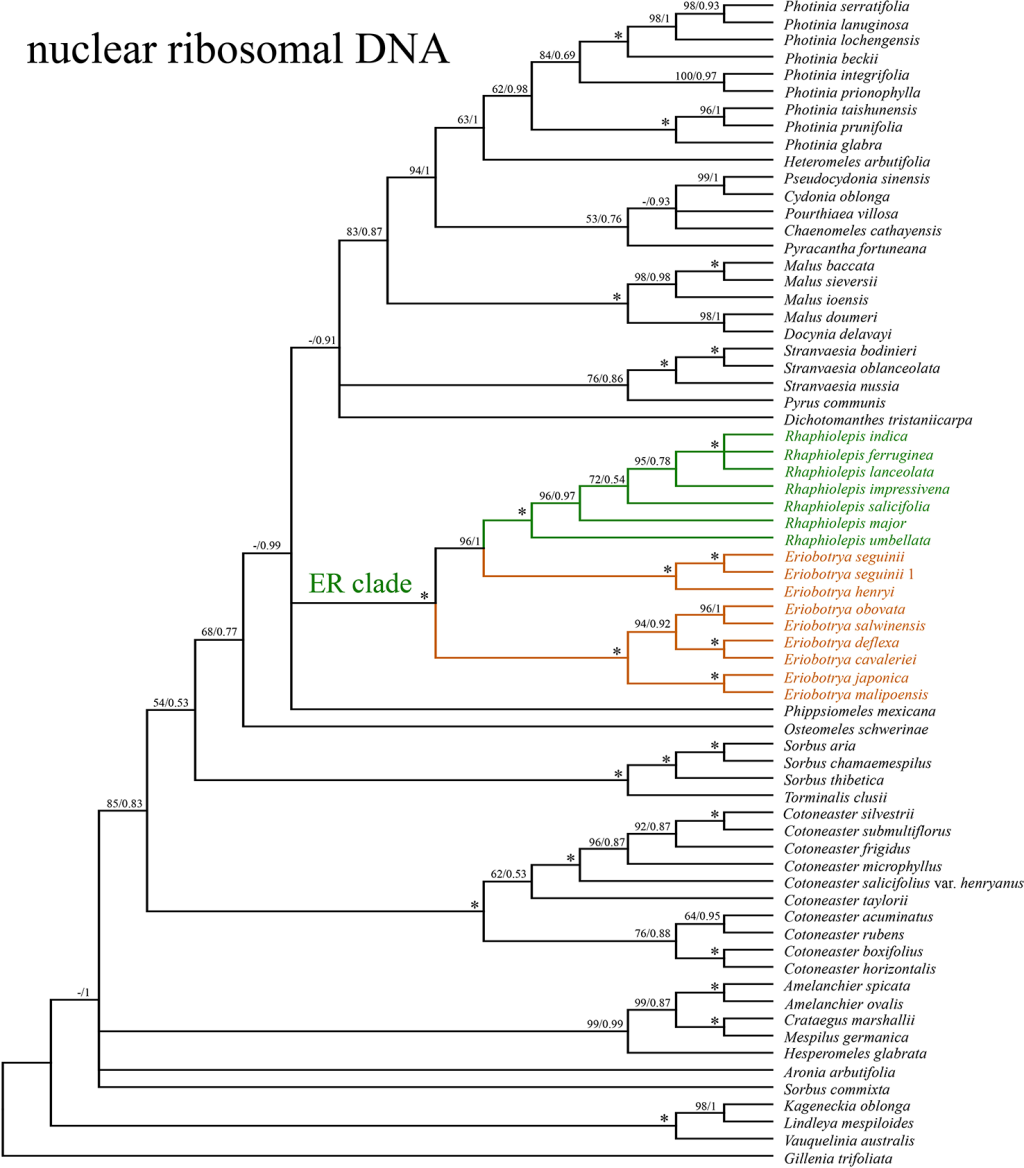
The phylogenetic relationships between *Eriobotrya* and *Rhaphiolepis* in the framework of Maleae resolved by Bayesian inference of the sequences of the nuclear ribosomal DNA (nrDNA) repeats. Numbers associated with the branches are ML bootstrap value (BS) and BI posterior probabilities (PP), and asterisks (*) indicate bootstrap support/posterior probability of 100/1.00.

**Figure 5:**
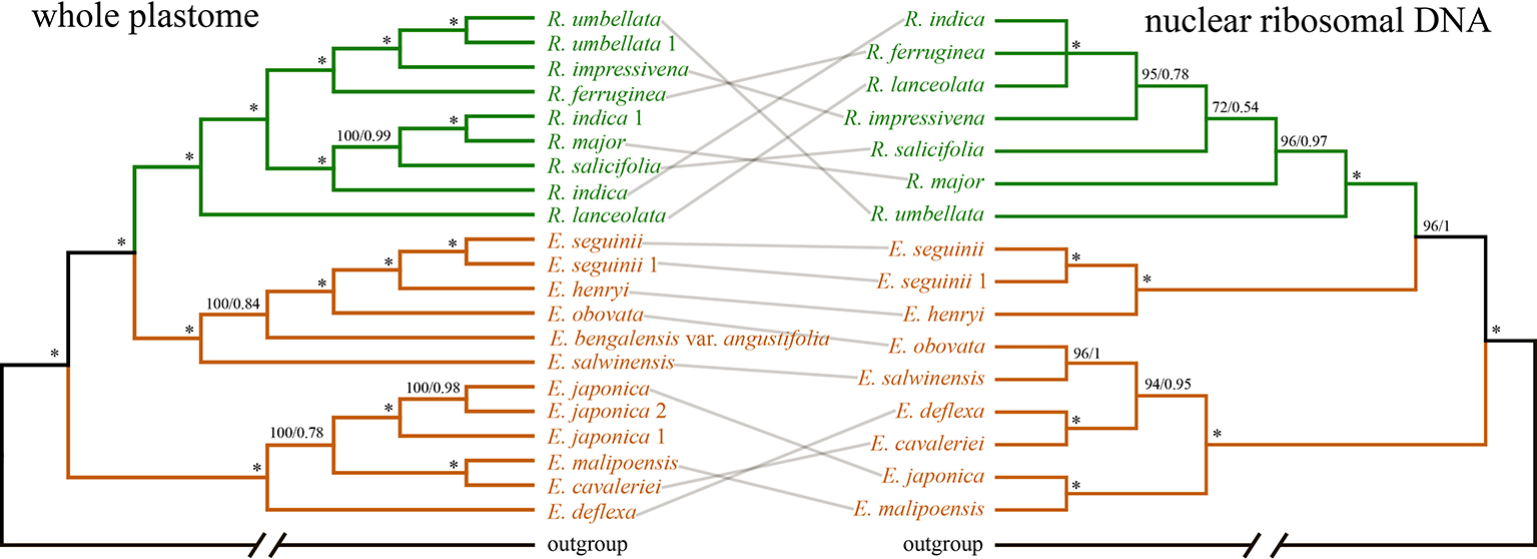
COMPARISONS of the phylogenies based on Bayesian inference of the whole plastid genome (WP: left) and nuclear ribosomal DNA (nrDNA) repeats (right). Branches are colored by genera. Numbers associated with the branches are ML bootstrap value (BS) and BI posterior probabilities (PP), and asterisks (*) indicate bootstrap support/posterior probability of 100/1.00.

The concatenated nrDNA data (six regions, i.e. partial ETS, 18S, ITS1, 5.8S, ITS2, and 26S) generated an aligned matrix of 6,361 bp in length. Each region of nrDNA was treated as a data block, and these six sets of sites were partitioned into five subsets with each corresponding models of molecular evolution for the subsequent ML and BI analyses. The ML and BI analyses had nearly the same topology (**Figure 4**). The ER clade was well-supported in the nrDNA tree (BS = 100, PP = 1), however, its phylogenetic position was not resolved (**Figure 4**). Two species of *Eriobotrya* (*E. henryi* and *E. seguinii*) were strongly supported to be sister to all *Rhaphiolepis* species sampled here (BS = 90, PP = 0.96), and then together they were sister to a clade of the other species of *Eriobotrya* (*E. cavaleriei*, *E. deflexa*, *E. japonica*, *E. malipoensis*, *E. obovata*, and *E. salwinensis*) (BS = 100, PP = 1). The topology of the ER clade generated from the nrDNA dataset showed strong conflicts with that from the plastome dataset (**Figure 5**).

## Discussion

### Enigmatic Phylogenetic Position of the *Eriobotrya-Rhaphiolepis* Clade

Our phylogenetic results based on the whole chloroplast genome and entire nrDNA repeats strongly supported the monophyly of the *Eriobotrya-Rhaphiolepis* complex (**Figure 3** and **Supplementary Figure 2**). The ER clade was first reported by Campbell et al. (2007) based on phylogenetic analyses using six cpDNA regions, and this relationship was supported by subsequent studies using chloroplast regions or even plastomes, but with limited taxon sampling (Lo and Donoghue, 2012; Zhang et al.,2017; Sun et al., 2018; Liu et al., 2019). In addition, *Eriobotrya* and *Rhaphiolepis* have also been supported to form a clade based on nuclear data, e.g., *GBSSI-1A* and nrITS plus *GBSSI-2B* sequences (Campbell et al., 2007), as well as nuclear *Adh* sequences (Yang et al., 2012). Although the topology of [*Eriobotrya*, (*Rhaphiolepis*, *Vauquelinia*)] was reported by Campbell et al. (1995) using nrITS and a small portion of the 5.8S sequences, all the subsequent studies based on the nrITS sequences supported the close relationship between *Eriobotrya* and *Rhaphiolepis* (Campbell et al., 2007; Li et al., 2009; Li et al., 2012; Lo and Donoghue, 2012) instead of between *Rhaphiolepis* and *Vauquelinia* (Campbell et al., 1995). Our nrDNA tree showed a distant relationship between *Vauquelinia* and the ER clade. In addition, *Vauquelinia* can be easily distinguished from the members of the ER clade morphologically by the former's dry capsular fruits, winged seeds with endosperms, and different wood ray anatomy (Zhang, 1992). Morphologically the ER clade is supported by two synapomorphies: the rounded or widely elliptic cross-section of seeds and the absence of endosperm (Aldasoro et al., 2005).

The ML and BI analyses based on the WP and CDS dataset strongly supported the sister relationship between the ER clade and *Heteromeles*, then together they were sister to *Photinia*. Previous studies based on the limited chloroplast regions or genomes have shown the uncertain placement of the ER clade. The clade may be sister to the [(*Aria*, *Heteromeles*) *Cotoneaster*] clade (Campbell et al., 2007), or *Cotoneaster* (Lo and Donoghue, 2012), or the [(*Cotoneaster*, *Stranvaesia*), (*Heteromeles*, *Photinia*)] clade (Sun et al., 2018), or the [*Photinia* (*Cotoneaster*, *Heteromeles*)] clade (Zhang et al., 2017; Liu et al., 2019). Such uncertainties may be due to the limited markers and/or samples used in the previous studies, or extinctions of the closest relatives of the ER clade. The ER clade was found to be closely related to the small North American genus *Heteromeles* based on our plastome data (**Figure 3** and **Supplementary Figure 2**). But we will test this relationship in our further analyses of Maleae.

By contrast, the ML and BI analyses based on the nrDNA dataset did not resolve the phylogenetic position of the ER clade. The ML result supported that the ER clade was sister to a large clade that includes *Chaenomeles*, *Cydonia*, *Dichotomanthes*, *Docynia*, *Heteromeles*, *Malus*, *Phippsiomeles*, *Photinia*, *Pourthiaea*, *Pseudocydonia*, *Pyracantha*, *Pyrus*, and *Stranvaesia* (**Figure 4**). The BI result did not resolve the relationships among the ER clade, the Central American *Phippsiomeles*, and the remaining genera (*Chaenomeles*, *Cydonia*, *Dichotomanthes*, *Docynia*, *Heteromeles*, *Malus*, *Photinia*, *Pourthiaea*, *Pseudocydonia*, *Pyracantha*, *Pyrus*, and *Stranvaesia*). Campbell et al. (1995) recovered the ER clade as well as *Vauquelinia* as forming the basalmost clade of Maleae using ITS sequences, and this result was supported by the cladistic analysis using 16 morphological and anatomical characters (Aldasoro et al., 2005). While the ER clade was placed either as sister to *Pyrus* using *GBSSI-1A* sequences or as a weakly supported sister group with *Chaenomeles* using *GBSSI-2B* and nrITS plus *GBSSI-2B* sequences (Campbell et al., 2007). The ER clade was moderately supported to be sister to *Osteomeles* and *Stranvaesia* using entire nrDNA repeats (Liu et al., 2019). Based on the transcriptome data, Xiang et al. (2017) placed the ER clade as sister to a large clade that includes *Chaenomeles*, *Cydonia*, *Docynia*, *Eriolobus*, *Malus*, *Photinia*, *Pseudocydonia*, *Pyrus*, *Sorbus*, and *Stranvaesia*, and this relationship does not conflict with our result. The ER clade is largely distributed in the warm temperate to subtropical and tropical regions of China, Indochina and the Malesian region, whereas most other Asian Maleae including its closely related genera are well developed in cool temperate and warm temperate regions, extending to subtropical regions of the Northern Hemisphere. Fossils of the ER clade were reported from northern China and northeastern Siberia in the Miocene when the earth was warmer (Hsu and Chaney, 1940; Baikovskaja, 1974).

The ER clade can be easily distinguished from other genera of Maleae. They have evergreen, coriaceous leaves with unlobed, entire to finely or coarsely serrate margins (Robertson et al., 1992), and semi-inferior to inferior ovaries (Rohrer et al., 1991; and its seed morphology is unique with the lack of endosperm and proportionally larger in size with a rounded or wide-elliptic cross-section (Rohrer et al., 1991; Aldasoro et al., 2005). The clade was placed as sister to all pome-bearing members of Maleae based on morphological characters (Aldasoro et al., 2005), supporting its isolated position. The incongruent positions of the ER clade based on our plastome and the nrDNA data may also point to the potential impact of hybridization. So the ER clade remains an enigmatic position within Maleae.

### Paraphyly of *Eriobotrya*

Our results strongly supported the paraphyly of *Eriobotrya*, with *Rhaphiolepis* nested within it, based on the plastome as well as the nrDNA datasets. The paraphyly of *Eriobotrya* was never reported in any previous studies. Yang et al. (2012) sampled only one species of *Rhaphiolepis* (*R. indica*) in their preliminary phylogenetic analyses of *Eriobotrya* using the nuclear *Adh* sequences. *Rhaphiolepis indica* was shown to be sister to a subclade of *Eriobotrya* (*E. bengalensis* Hook.f., *E. prinoides* Rehder & E.H.Wilson f. *angustifolia* J.E.Vidal, *E. henryi*, and *E. seguinii*), and this clade was then sister to another subclade of nine *Eriobotrya* species, showing the biphyly of *Eriobotrya*, with an extremely limited sampling of *Rhaphiolepis*. Yang et al. (2012) never discussed the nonmonophyly of *Eriobotrya* nor its taxonomic implications. Due to the general limited taxon sampling of *Eriobotrya* and *Rhaphiolepis*, most previous studies only emphasized the close relationships between these two genera (Campbell et al., 2007; Lo and Donoghue, 2012; Xiang et al., 2017; Yang et al., 2017; Zhang et al., 2017), but their data were insufficient in addressing the phylogenetic relationships between the two genera.

**Figure 6:**
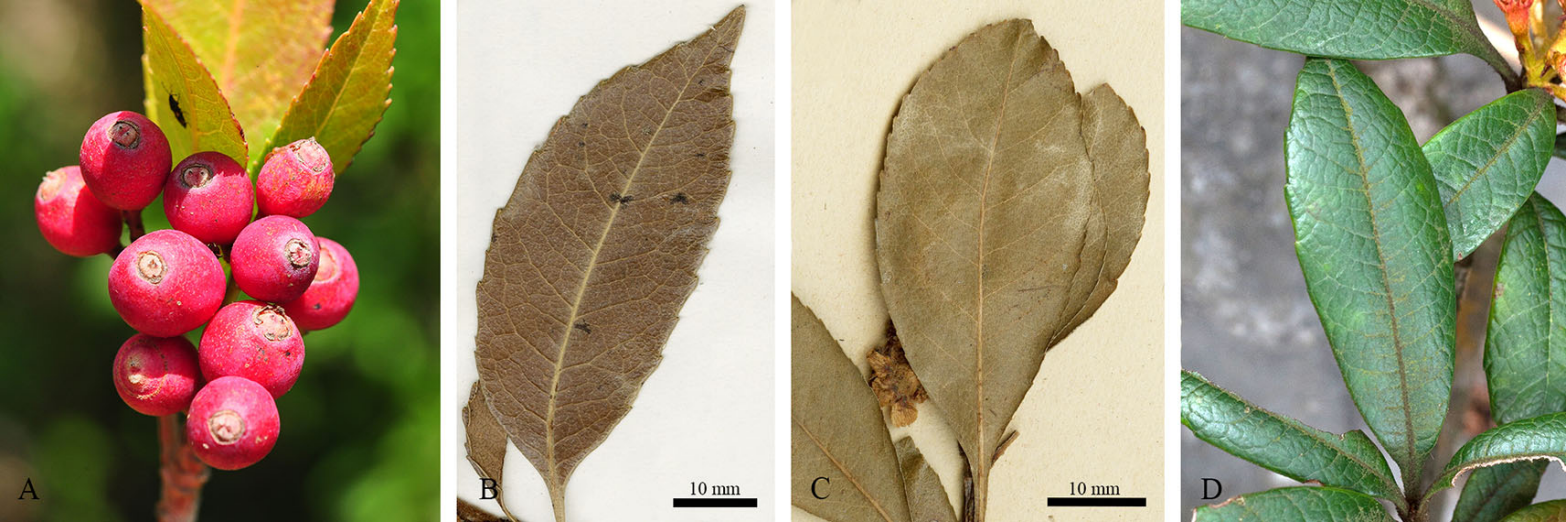
The caducous sepals (**A**, *Eriobotrya henryi* from Yunnan, China); curved lateral veins of leaves (camptodroumous) in *Eriobotrya* (**B**, *E. henryi* A[bardoce 00063057] and **C**, *E. seguinii*); the lateral veins terminate at the leaf margin (craspedodromous) in *Rhaphiolepis ferruginea* F.P.Metcalf (Guangxi, China). Photo credits: **(A)**, Jian Huang; **(D)**, Bin-Bin Liu.

Our study represents the first phylogenetic analysis that was designed to test the monophyly of *Eriobotrya* and *Rhaphiolepis* with the taxon sampling representing their respective morphological and geographic diversity. The paraphyly of *Eribototrya* revealed in our plastid and nrDNA supports the merge of *Eriobotrya* and *Rhaphiolepis* into one genus. *Eriobotrya* was thought to be easily distinguished from *Rhaphiolepis* by the former's persistent sepals (vs. caducous sepals in *Rhaphiolepis*) and the excurrent lateral veins of leaves (vs. curved lateral veins in *Rhaphiolepis*). However, these two characters were not always stable to be used to distinguish *Eriobotrya* and *Rhaphiolepis*. For example, based on our field observations and herbarium studies in PE and US, the sepals of *Eriobotrya henryi* are obviously caducous (**Figure 6A**) and the lateral veins of leaves in *E. henryi* and *E. seguinii* are curved (**Figures 6B, C**), furthermore, the lateral veins of *Rhaphiolepis* sometimes terminate at the leaf margins (**Figure 6D**). Treating the ER clade as one genus is also supported by two synapomorphies: the presence of rounded or widely elliptic seeds and the absence of endosperm). Geographically, these two genera overlap broadly in East and Southeast Asia (**Figure 1**). The phylogenetic, morphological and geographic evidence all supports merging *Eriobotrya* into *Rhaphiolepis*, which has the nomenclatural priority. Nevertheless, describing the clade II of **Figures 3** as a new genus may seem a likely alternative solution to ensure the monophyly of each genus, however, the phylogenetic incongruence (e.g., between *E. obovata* and *E. salwinensis*) in the chloroplast and nuclear trees is consistent with the extensive gene flow between clades II and III (see below). It thus seems not a good taxonomic solution to segregate clade II of *Eriobotrya* into a separate genus.

We detected topological incongruence within the expanded *Rhaphiolepis* between the chloroplast and nrDNA trees (**Figure 5**). Processes that might explain the incongruence between chloroplast and nuclear phylogenies include incomplete lineage sorting, allopolyploidy, and hybridization (Rieseberg and Soltis, 1991; Mckinnon et al., 2001; Zou and Ge, 2008). Allopolyploidy has never been reported in *Eriobotrya* except for the cultivars of *E. japonica* (Mehra et al., 1973; Singhal et al., 1990; Chen, 1993; Liang et al., 1999; Liang et al., 2001; Yahata et al., 2005; Zhang et al., 2012; Li et al., 2013a), and this mechanism may be excluded for explaining the conflicts between the plastid and nrDNA trees. Lineage sorting could also result in incongruence between chloroplast and nuclear topologies, however, it is very difficult to distinguish hybridization from incomplete lineage sorting (Joly et al., 2009). Hybridization has been shown to be very common in Maleae (Robertson et al., 1991; Lo and Donoghue, 2012; Liu et al., 2019), and artificial hybrids were reported even between *Eriobotrya* and *Rhaphiolepis* (Coombes and Robertson, 2008). The conflicts shown in our results likely reflect frequent hybridization events in the evolutionary history of the expanded *Rhaphiolepis*. The extent and impact of hybridizations in Maleae will need to be further analyzed using next-generation sequencing and genomic tools (Zimmer and Wen, 2015). We will discuss the evolutionary events, involving hybridization, chloroplast capture, introgression, and/or allopolyploidy that occurred in the expanded *Rhaphiolepis* with a comprehensive sampling in a follow-up paper.

### Taxonomy

***Rhaphiolepis*** Lindl., Bot. Reg. 6: t. 468 (1820)≡*Rhaphiolepis* Poir., Dict. Sci. Nat., ed. 2. [F. Cuvier] 45: 314. 1827. Type: *Rhaphiolepis indica* (L.) Lindl. ex Ker Gawl.≡*Crataegus indica* L. = *Eriobotrya* Lindl., Trans. Linn. Soc. London 13: 96, 102. 1821. **syn. nov.**

Trees, small trees, or shrubs, unarmed, evergreen. Leaves simple, penninerved; stipules on the extreme base of petiole, free, rarely intrapetiolarly connate. Petiole present, venation craspedodromous or camptodromous, margin serrate or entire; stipules caducous, subulate. Inflorescences in terminal racemes, panicles, or compound racemes, many flowered. Hypanthium campanulate, cupular, tubular, or obconical, the free part inside lined with an intrastaminal disk, open at top. Sepals 5, persistent or caducous. Petals 5, white, yellow, or pink, obovate or orbicular, base clawed. Stamens 15–20(-40). Ovary inferior, carpels 2–5, ventrally and laterally connate (in upper part ventrally free), completely connate with each other and dorsally adnate to the hypanthium, the hairy apex exposed; ovules normally 2 per carpel, rarely more; styles 2–5, connate at base and often pubescent; stigma truncate. Fruit a pome, yellowish, yellowish red, brown, dark purplish-brown, bluish, or purplish-black, subglobose, globose, or obovate, fleshy or dry, flesh mostly of hypanthial origin, sclereids absent or present, endocarp (core) thin, membranous. Seeds 1–3, large, with a thin but firm testa; endosperm absent, cotyledons thick. 2*n* = 34.

About 46 species (Vidal, 1965; Vidal, 1968; Vidal, 1970; Kalkman, 1973; Lu et al., 2003; Kalkman, 2004) in East & Southeast Asia and the Himalayas, south to Borneo and Sumatra.

Below we transfer all taxa of *Eriobotrya* to *Rhaphiolepis* and make the nomenclatural changes.

1. ***Rhaphiolepis angustissima*** (Hook.f.) B.B.Liu & J.Wen, **comb. nov.**≡*Eriobotrya angustissima* Hook.f., Fl. Brit. India [J. D. Hooker] 2(5): 372. 1878≡*Pyrus angustissima* (Hook.f.) M.F.Fay & Christenh., Global Fl. 4: 95. 2018.

Distribution: India (Mt. Khasia); South Vietnam.

2. ***Rhaphiolepis balgooyi*** (K.M.Wong & Ent) B.B.Liu & J.Wen, **comb. nov.**≡*Eriobotrya balgooyi* K.M.Wong & Ent, Pl. Ecol. Evol. 147(1): 136. 2014.

Distribution: Malaysia (Borneo on Mt. Kinabalu and Mt. Tambuyukon).

3. ***Rhaphiolepis bengalensis*** (Roxb.) B.B.Liu & J.Wen, **comb. nov.**≡*Mespilus bengalensis* Roxb., Hort. Bengal. 38; Fl. Ind. (ed. 1832) 2: 510. 1832≡*Eriobotrya bengalensis* Hook.f., Fl. Brit. India [J. D. Hooker] 2(5): 371. 1878.

= *Alsodeia grandis* Miq., Fl. Ned. Ind., Eerste Bijv. 3: 391. 1861.

= *Eriobotrya tinctoria* Kurz, Prelim. Rep. For. Veg. Pegu, App. B. 48. 1875, in clavi.

Distribution: widely distributed from East Himalaya (Sikkim, Assam) through Bangladesh (Chittagong) to Myanmar, Laos, Cambodia, S. Vietnam, Malay Peninsula, Sumatra, and Borneo.

4. ***Rhaphiolepis cavaleriei*** (H.Lév.) B.B.Liu & J.Wen, **comb. nov.**≡*Hiptage cavaleriei* H.Lév., Repert. Spec. Nov. Regni Veg. 10: 372. 1912≡*Eriobotrya cavaleriei* (H.Lév.) Rehder, J. Arnold Arbor. 13: 307. 1932≡*Pyrus athenae* M.F.Fay & Christenh., Global Fl. 4: 96. 2018.

= *Eriobotrya brackloi* Hand.-Mazz., Anz. Akad. Wiss. Wien, Math.-Naturwiss. Kl. 59: 102. 1922≡*Eriobotrya cavaleriei* (H.Lév.) Rehder var. *brackloi* (Hand.-Mazz.) Rehder, J. Arnold Arbor. 13(3): 308. 1932.

= *Eriobotrya brackloi* Hand.-Mazz. var. *atrichophylla* Hand.-Mazz., Anz. Akad. Wiss. Wien, Math.-Naturwiss. Kl. 59: 103. 1922.

= *Eriobotrya grandiflora* Rehder & E.H.Wilson, Pl. Wilson. (Sargent) 1(2): 193. 1912≡*Eriobotrya deflexa* (Hemsl.) Nakai var. *grandiflora* (Rehder & E.H.Wilson) Nakai, J. Arnold Arbor. 5(2): 72. 1924.

Distribution: China (Fujian, Guangdong, Guangxi, Guizhou, Hubei, Hunan, Jiangxi, Sichuan); North Vietnam (Hòa Bình, Lao Cai).

5. ***Rhaphiolepis condaoensis*** (X.F.Gao, Idrees & T.V.Do) B.B.Liu & J.Wen, **comb. nov.**≡*Eriobotrya condaoensis* X.F.Gao, Idrees & T.V.Do, Phytotaxa 365(3): 290. 2018.

Distribution: Southeast Vietnam (Ba Ria-Vung Tau: Con Dao National Park).

6. ***Rhaphiolepis × daduheensis*** (H.Z.Zhang ex W.B.Liao, Q.Fan & M.Y.Ding) B.B.Liu & J.Wen, **comb. nov.**≡*Eriobotrya × daduheensis* H.Z.Zhang ex W.B.Liao, Q.Fan & M.Y.Ding, Phytotaxa 212(1): 97. 2015.

Distribution: As a putative natural hybrid between *Rhaphiolepis loquata* (= *Eriobotrya japonica*) and *R. prinoides* (= *E. prinoides*), this species is restricted to Daduhe River Basin in Sichuan, China (Ding et al., 2015).

7. ***Rhaphiolepis deflexa*** (Hemsl.) B.B.Liu & J.Wen, **comb. nov.**≡*Photinia deflexa* Hemsl., in Ann. Bot. ix. 153. 1895≡*Eriobotrya deflexa* (Hemsl.) Nakai, Bot. Mag. (Tokyo) 30: 18, in adnot. 1916.

= *Photinia buisanensis* Hayata, Icon. Pl. Formosan. 3: 100. 1913≡*Eriobotrya deflexa* (Hemsl.) Nakai f. *buisanensis* (Hayata) Nakai, Bot. Mag. (Tokyo) 30(349): 18. 1916≡*Eriobotrya buisanensis* (Hayata) Kaneh., Formosan Trees 218. 1918≡*Eriobotrya deflexa* Nakai var. *buisanensis* (Hayata) Hayata, Catal. Governm. Herb. Formos. 246. 1930≡*Eriobotrya buisanensis* (Hayata) Makino & Nemoto, Fl. Japan., ed. 2 (Makino & Nemoto) 464. 1931.

= *Eriobotrya deflexa* Nakai var. *koshunensis* Kaneh. & Sasaki, Catal. Gov't Herb. Formosa 246. 1930≡*Eriobotrya deflexa* Nakai f. *koshunensis* (Kaneh. & Sasaki) H.L.Li, Lloydia 14(4): 232. 1951.

Distribution: China (Guangdong, Hainan, Taiwan); Vietnam (Nha Trang).

8. ***Rhaphiolepis dubia*** (Lindl.) B.B.Liu & J.Wen, **comb. nov.**≡*Photinia dubia* Lindl., Trans. Linn. Soc. London 13(1): 104, t. 10. 1821≡*Eriobotrya dubia* (Lindl.) Decne., in Nouv. Arch. Mus. Par. Ser. I, x. 145. 1874.

= *Mespilus tinctoria* D.Don, Prodr. Fl. Nepal. 238. 1825.

Distribution: Bhutan; India (Sikkim); Myanmar (Kachin, Mandalay, Shan); Nepal.

9. ***Rhaphiolepis elliptica*** (Lindl.) B.B.Liu & J.Wen, **comb. nov.**≡*Eriobotrya elliptica* Lindl., Trans. Linn. Soc. London 13(1): 102. 1821≡*Cotoneaster ellipticus* (Lindl.) Hort ex Loudon, Encyc. Pl. 1208≡*Pyrus elliptica* (Lindl.) M.F.Fay & Christenh., Global Fl. 4: 102. 2018.

= *Mespilus cuila* Buch.-Ham. ex D.Don, Prodr. Fl. Nepal. 238. 1825.

Distribution: China (Tibet); Nepal (Narainhetty).

10. ***Rhaphiolepis elliptica*** (Lindl.) B.B.Liu & J.Wen var. ***petelotii*** (J.E.Vidal) B.B.Liu & J.Wen, **comb. nov.**≡*Eriobotrya elliptica* Lindl. var. *petelotii* J.E.Vidal, Adansonia sér. 2, 5: 552. 1965.

Distribution: N Vietnam (Lao Cai).

11. ***Rhaphiolepis fulvicoma*** (Chun ex W.B.Liao, F.F.Li & D.F.Cui) B.B.Liu & J.Wen, **comb. nov.**≡*Eriobotrya fulvicoma* Chun ex W.B.Liao, F.F.Li & D.F.Cui, Ann. Bot. Fenn. 49(4): 264. 2012.

Distribution: China (Guangdong).

12. ***Rhaphiolepis glabrescens*** (J.E.Vidal) B.B.Liu & J.Wen, **comb. nov.**≡*Eriobotrya glabrescens* J.E.Vidal, Adansonia sér. 2, 5: 554. 1965≡*Pyrus serpentae* M.F.Fay & Christenh., Global Fl. 4: 121. 2018.

Distribution: North Myanmar (Triangle, Centre Ouest, Khai Yang).

13. ***Rhaphiolepis glabrescens*** (J.E.Vidal) B.B.Liu & J.Wen var. ***victoriensis*** (J.E.Vidal) B.B.Liu & J.Wen, **comb. nov.**≡*Eriobotrya glabrescens* J.E.Vidal var. *victoriensis* J.E.Vidal, Adansonia sér. 2, 5: 555. 1965.

Distribution: North Mayanmar (Centre Ouest: Mt Victoria).

14. ***Rhaphiolepis henryi*** (Nakai) B.B.Liu & J.Wen, **comb. nov.**≡*Eriobotrya henryi* Nakai, J. Arnold Arbor. 5: 70. 1924≡*Pyrus henryi* (Nakai) M.F.Fay & Christenh., Global Fl. 4:106. 2018.

Distribution: China (Guizhou, Yunnan); Myanmar (Pyin Oo Lwin).

15. ***Rhaphiolepis hookeriana*** (Decne.) B.B.Liu & J.Wen, **comb. nov.**≡*Eriobotrya hookeriana* Decne., Nouv. Arch. Mus. Par. Ser. I 10:146. 1874≡*Pyrus hookeriana* (Decne.) M.F.Fay & Christenh., Global Fl. 4: 107. 2018.

Distribution: Bhutan; India (Sikkim).

16. ***Rhaphiolepis herae*** (M.F.Fay & Christenh.) B.B.Liu & J.Wen, **comb. nov.**≡*Eriobotrya latifolia* Hook.f., Fl. Brit. India [J. D. Hooker] 2(5): 370. 1878≡*Pyrus herae* M.F.Fay & Christenh., Global Fl. 4: 106. 2018.

Distribution: Myanmar (Kayin, Taninthayi).

17. ***Rhaphiolepis longifolia*** (Decne.) B.B.Liu & J.Wen, **comb. nov.**≡*Photinia longifolia* Decne., in Nouv. Arch. Mus. Par. Ser. I 10: 142. 1874≡*Eriobotrya longifolia* (Decne.) Hook.f., Fl. Brit. India [J. D. Hooker] 2(5): 370. 1878.

Distribution: Bangladesh (East Bengal).

18. ***Rhaphiolepis loquata*** B.B.Liu & J.Wen, **nom. nov.**≡*Mespilus japonica* Thunb., Fl. Jap. (Thunberg) 206. 1784≡*Eriobotrya japonica* (Thunb.) Lindl., Trans. Linn. Soc. London 13: 102. 1821≡*Photinia japonica* Benth. & Hook.f. ex Asch. & Schweinf., Mém. Inst. Égypt. [Illustr. Fl. Egypt.] 73. 1887.

= *Crataegus bibas* Lour., Fl. Cochinch. 1: 319. 1790≡*Pyrus bibas* (Lour.) M.F.Fay & Christenh., Global Fl. 4: 98. 2018.

Distribution: Native in Chongqing (Nanchuan) and Hubei (Yichang) of China. As an economically important fruit, this species has been widely cultivated in central & south China, as well as in Japan, Korea, India, and some countries in Southeast Asia.

Note: The species epithet has been pre-occupied by *Rhaphiolepis japonica* Siebold & Zucc. (Siebold and Zuccarini, 1841), so a new name is needed for this taxon (Turland et al., 2018). The epithet “loquata” is derived from the English name loquat.

19. ***Rhaphiolepis macrocarpa*** (Kurz) B.B.Liu & J.Wen, **comb. nov.**≡*Eriobotrya macrocarpa* Kurz, J. Asiat. Soc. Bengal, Pt. 2, Nat. Hist. 41(4): 306. 1872.

Distribution: Myanmar (Bago, Mandalay).

20. ***Rhaphiolepis malipoensis*** (K.C.Kuan) B.B.Liu & J.Wen, **comb. nov.**≡*Eriobotrya malipoensis* K.C.Kuan, Acta Phytotax. Sin. 8(3): 231. 1963≡*Pyrus malipoensis* (K.C.Kuan) M.F.Fay & Christenh., Global Fl. 4: 111. 2018.

Distribution: China (SE Yunnan).

21. ***Rhaphiolepis merguiensis*** (J.E.Vidal) B.B.Liu & J.Wen, **comb. nov.**≡*Eriobotrya merguiensis* J.E.Vidal, Adansonia sér. 2, 5: 563. 1965.≡*Pyrus merguiensis* (J.E.Vidal) M.F.Fay & Christenh., Global Fl. 4: 112. 2018.

Distribution: Myanmar (Mergui Archipelago, Taninthayi).

22. ***Rhaphiolepis oblongifolia*** (Merr. & Rolfe) B.B.Liu & J.Wen, **comb. nov.**≡*Eriobotrya oblongifolia* Merr. & Rolfe, Philipp. J. Sci., C 3: 102. 1908.

Distribution: the Philippines (Mindanao).

23. ***Rhaphiolepis obovata*** (W.W.Sm.) B.B.Liu & J.Wen, **comb. nov.**≡*Eriobotrya obovata* W.W.Sm., Notes Roy. Bot. Gard. Edinburgh 10: 29. 1917≡*Pyrus obovata* (W.W.Sm.) M.F.Fay & Christenh., Global Fl. 4: 114. 2018.

Distribution: China (C Yunnan).

24. ***Rhaphiolepis petiolata*** (Hook.f.) B.B.Liu & J.Wen, **comb. nov.**≡*Eriobotrya petiolata* Hook.f., Fl. Brit. India [J. D. Hooker] 2(5): 370. 1878≡*Pyrus petiolata* (Hook.f.) M.F.Fay & Christenh., Global Fl. 4: 115. 2018.

Distribution: Bangladesh (Chittagong); Bhutan; India (Khasia, Sikkim); Myanmar (Chin).

25. ***Rhaphiolepis platyphylla*** (Merr.) B.B.Liu & J.Wen, **comb. nov.**≡*Eriobotrya platyphylla* Merr., Brittonia 4(1): 80. 1941≡*Pyrus platyphylla* (Merr.) M.F.Fay & Christenh., Global Fl. 4: 116. 2018.

Distribution: Myanmar (Kachin).

26. ***Rhaphiolepis poilanei*** (J.E.Vidal) B.B.Liu & J.Wen, **comb. nov.**≡*Eriobotrya poilanei* J.E.Vidal, Adansonia sér. 2, 5: 557. 1965≡*Pyrus poilanei* (J.E.Vidal) M.F.Fay & Christenh., Global Fl. 4: 116. 2018.

Distribution: Vietnam (Haut-Donnai).

27. ***Rhaphiolepis prinoides*** (Rehder & E.H.Wilson) B.B.Liu & J.Wen, **comb. nov.**≡*Eriobotrya prinoides* Rehder & E.H.Wilson, Pl. Wilson. (Sargent) 1(2): 194. 1912≡*Pyrus prinoides* (Rehder & E.H.Wilson) M.F.Fay & Christenh., Global Fl. 4: 116. 2018.

Distribution: China (Sichuan, Yunnan); Laos.

28. ***Rhaphiolepis prinoides*** (Rehder & E.H.Wilson) B.B.Liu & J.Wen var. ***laotica*** (J.E.Vidal) B.B.Liu & J.Wen, **comb. nov.**≡*Eriobotrya prinoides* Rehder & E.H.Wilson var. *laotica* J.E.Vidal, Adansonia sér. 2, 5: 573. 1965.

Distribution: Laos (Xièng Khouang).

29. ***Rhaphiolepis salwinensis*** (Hand.-Mazz.) B.B.Liu & J.Wen, **comb. nov.**≡*Eriobotrya salwinensis* Hand.-Mazz., Symb. Sin. Pt. 7(3): 475. 1933≡*Pyrus salwinensis* (Hand.-Mazz.) M.F.Fay & Christenh., Global Fl. 4: 120. 2018.

Distribution: China (NE Yunnan; Tibet); India; Myanmar.

30. ***Rhaphiolepis seguinii*** (H.Lév.) B.B.Liu & J.Wen, **comb. nov.**≡*Symplocos seguinii* H.Lév., Repert. Spec. Nov. Regni Veg. 10: 431. 1912≡*Eriobotrya seguinii* (H.Lév.) Cardot ex Guillaumin, Bull. Soc. Bot. France 71: 287, in obs. 1924.

= *Eriobotrya pseudorhaphiolepis* Cardot, Notul. Syst. (Paris) 3: 371. 1918.

Distribution: China (SW Guizhou, SE Yunnan).

31. ***Rhaphiolepis serrata*** (J.E.Vidal) B.B.Liu & J.Wen, **comb. nov.**≡*Eriobotrya serrata* J.E.Vidal, Adansonia sér. 2, 5: 558. 1965≡*Pyrus serrata* (J.E.Vidal) M.F.Fay & Christenh., Global Fl. 4: 121. 2018.

Distribution: China (Guangxi, Yunnan); Laos (Xièng Khouang).

32. ***Rhaphiolepis stipularis*** (Craib) B.B.Liu & J.Wen, **comb. nov.**≡*Eriobotrya stipularis* Craib, Bull. Misc. Inform. Kew 1929(4): 109. 1929≡*Pyrus stipularis* (Craib) M.F.Fay & Christenh., Global Fl. 4: 122. 2018.

Distribution: Cambodia; Thailand (Satun).

33. ***Rhaphiolepis tengyuehensis*** (W.W.Sm.) B.B.Liu & J.Wen, **comb. nov.**≡*Eriobotrya tengyuehensis* W.W.Sm., Notes Roy. Bot. Gard. Edinburgh 10: 30. 1917≡*Pyrus tengyuehensis* (W.W.Sm.) M.F.Fay & Christenh., Global Fl. 4: 123. 2018.

Distribution: China (NW Yunnan; Tibet); Myanmar (Kachin).

34. ***Rhaphiolepis wardii*** (C.E.C.Fisch.) B.B.Liu & J.Wen, **comb. nov.**≡*Eriobotrya wardii* C.E.C.Fisch., Bull. Misc. Inform. Kew 1929(6): 205. 1929≡*Pyrus alabaster* M.F.Fay & Christenh., Global Fl. 4: 94. 2018.

Distribution: Myanmar (Kachin, North Triangle).

35. ***Rhaphiolepis williamtelliana*** (M.F.Fay & Christenh.) B.B.Liu & J.Wen, **comb. nov.**≡*Eriobotrya fragrans* Champ., Hooker's J. Bot. Kew Gard. Misc. 4: 80. 1852≡*Pyrus williamtelliana* M.F.Fay & Christenh., Global Fl. 4: 126. 2018.

Distribution: China (Guangdong, Guangxi, Hainan, Hongkong, Tibet); Vietnam.

36. ***Rhaphiolepis williamtelliana*** (M.F.Fay & Christenh.) B.B.Liu & J.Wen var. ***furfuracea*** (J.E.Vidal) B.B.Liu & J.Wen, **comb. nov.**≡*Eriobotrya fragrans* Champ. ex Benth. var. *furfuracea* J.E.Vidal, Adansonia sér. 2, 5: 557. 1965.

Distribution: Vietnam (Nha Trang).

## Conclusion

Our phylogenetic analyses of Maleae using sequences of the whole chloroplast genomes and entire nrDNA repeats have strongly supported the monophyly of the *Eriobotrya*-*Rhaphiolepis* clade and the paraphyly of *Eriobotrya*, in which *Rhaphiolepis* was nested within it. Molecular, morphological, and geographic evidence supports merging these two genera into one genus, *Rhaphiolepis*. We herein have transferred taxa currently recognized in *Eriobotrya* to *Rhaphiolepis*, making 35 new combinations and a new name, *Rhaphiolepis loquata*, in this paper.

## Data Availability Statement

Publicly available datasets were analyzed in this study. This data can be found here: MN577874, MN577892, MN577875, MN577893, MN577873, MN577895, MN577863, MN577894, MN577872, MN577880, MN577877, MN577881, MN577882, MN577883, MN577884, MN577885, MN068252, MN068266, MN068248, MN577878, MN577889, MN577879, MN577890, MN577888, MN577891, MN577865, MN577871, MN577870, MN577866, MN577886, MN577864, MN577867, MN577887, MN577876, MN577868, MN068250, MN577908, MN577928, MN577909, MN577929, MN577907, MN577931, MN577896, MN577930, MN577905, MN577914, MN577911, MN577915, MN577916, MN577918, MN577919, MN577920, MN577923, MN577932, MN577906, MN577912, MN577925, MN577913, MN577926, MN577924, MN577927, MN577898, MN577904, MN577903, MN577899, MN577921, MN577897, MN577900, MN577922, MN577910, MN577901, MN577917.

## Author Contributions

B-BL, G-NL, D-YH, and JW designed the study. B-BL and G-NL conducted the experiments, analyzed the data, and drafted the manuscript. JW and D-YH provided suggestions on structuring the paper and the main points of the discussion, and revised the manuscript. All authors approved the final manuscript.

## Funding

This research is supported by the National Natural Science Foundation of China (Grant No 31620103902).

## Conflict of Interest

The authors declare that the research was conducted in the absence of any commercial or financial relationships that could be constructed as a potential conflict of interest.

## Supplementary Materials

Supplementary Figure 1 Gene map of the chloroplast genome of *Rhaphiolepis loquata* (*Eriobotrya japonica*). The genes inside and outside of the circle are transcribed in the clockwise and counterclockwise directions, respectively. Genes belonging to the different functional group are shown in different colors. The thick lines indicate the extent of the inverted repeats (IRa and IRb) that separate the genomes into small single-copy (SSC) and large single-copy (LSC) regions.

Supplementary Figure 2 The phylogenetic relationships between *Eriobotrya* and *Rhaphiolepis* in the framework of Maleae resolved by Bayesian inference of the coding sequences (CDS) of chloroplast genome. Numbers associated with the branches are ML bootstrap value (BS) and BI posterior probabilities (PP), and asterisks (*) indicate 100/1 support.

Supplementary Table 1 Accessions of Maleae used in this study. † and ‡ indicate that the samples were extracted from silica-gel dried leaves and herbarium specimens, respectively.
