# Supplementary figures and images for: Eriobotrya Belongs to Rhaphiolepis (Maleae, Rosaceae): Evidence From Chloroplast Genome and Nuclear Ribosomal DNA Data
Source: Front Plant Sci. 2020 Feb 7;10:1731. doi: 10.3389/fpls.2019.01731 (PMC7019104; doi:10.3389/fpls.2019.01731)

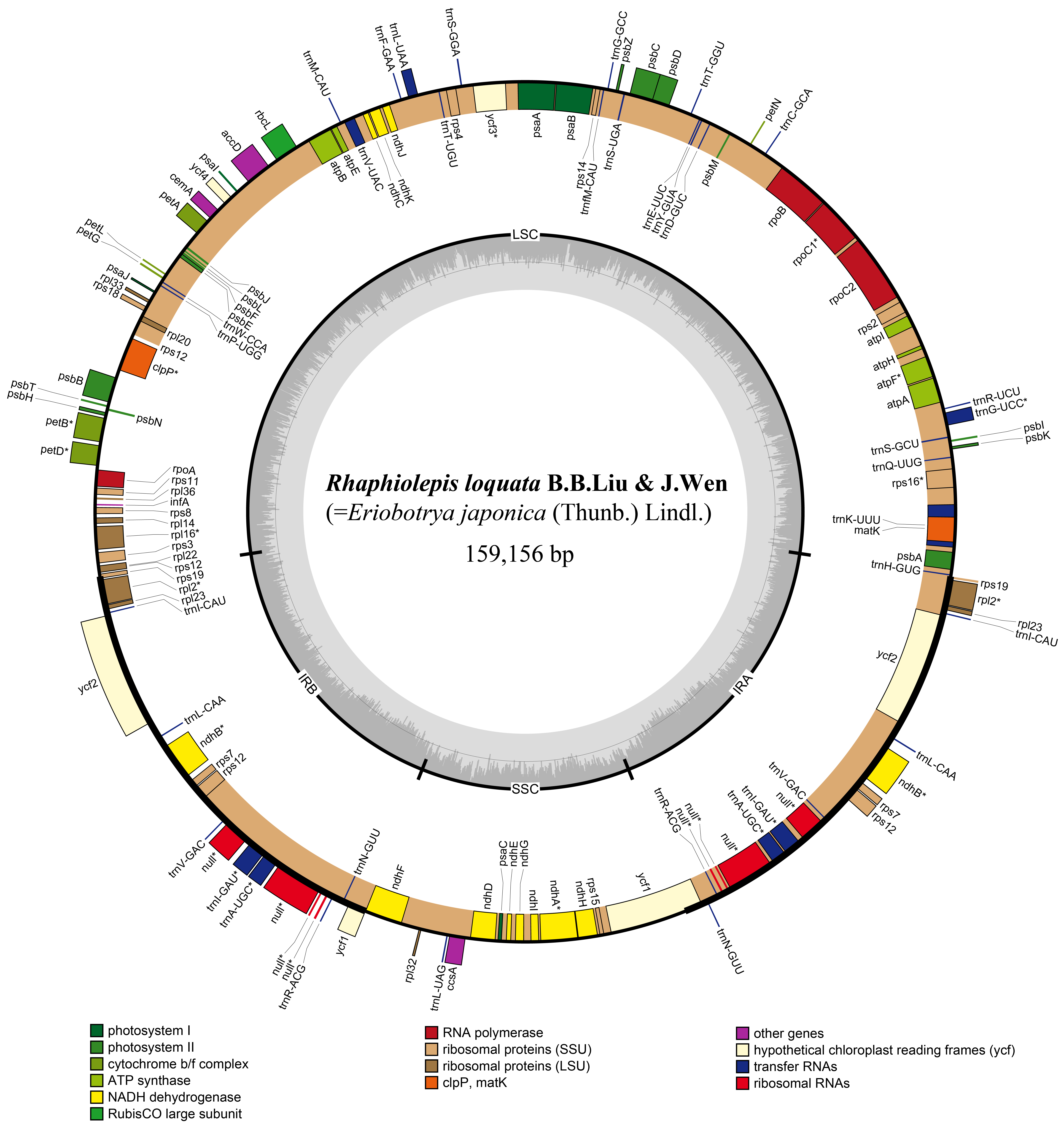

Supplement: Supplementary Figure 1 — Gene map of the chloroplast genome of Rhaphiolepis loquata (Eriobotrya japonica). The genes inside and outside of the circle are transcribed in the clockwise and counterclockwise directions, respectively. Genes belonging to the different functional group are shown in different colors. The thick lines indicate the extent of the inverted repeats (IRa and IRb) that separate the genomes into small single-copy (SSC) and large single-copy (LSC) regions. [file Image_1.jpg]

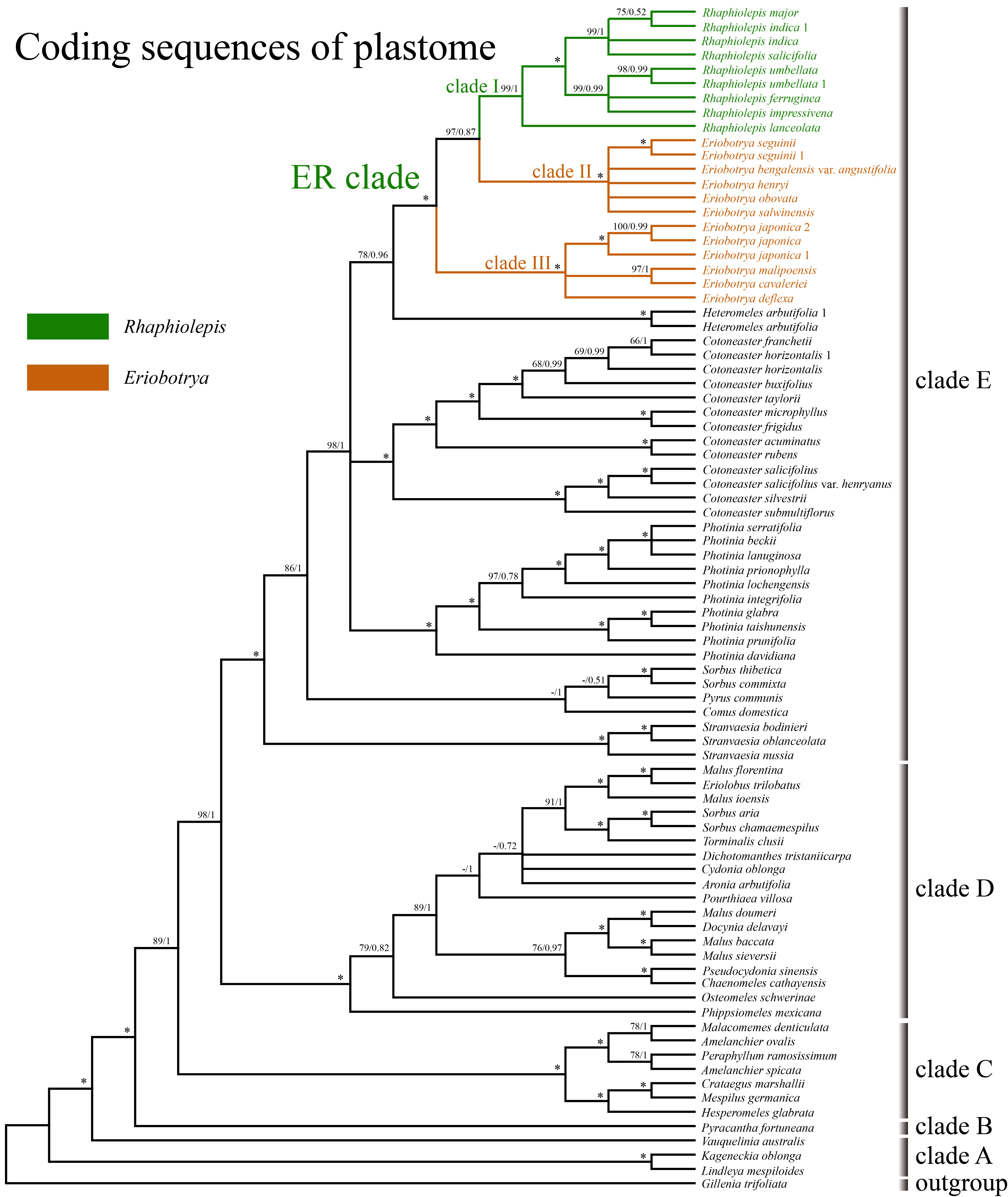

Supplement: Supplementary Figure 2 — The phylogenetic relationships between Eriobotrya and Rhaphiolepis in the framework of Maleae resolved by Bayesian inference of the coding sequences (CDS) of chloroplast genome. Numbers associated with the branches are ML bootstrap value (BS) and BI posterior probabilities (PP), and asterisks (*) indicate 100/1 support. [file Image_2.jpeg]
