# Supplementary material for: Eriobotrya Belongs to Rhaphiolepis (Maleae, Rosaceae): Evidence From Chloroplast Genome and Nuclear Ribosomal DNA Data
Source: Front Plant Sci. 2020 Feb 7;10:1731. doi: 10.3389/fpls.2019.01731 (PMC7019104; doi:10.3389/fpls.2019.01731)
Supplement: Supplementary Table 1 — Accessions of Maleae used in this study. † and ‡ indicate that the samples were extracted from silica-gel dried leaves and herbarium specimens, respectively. [file Table_1.docx]

**Supplementary Table 1**. Accessions of Maleae used in this study. † and ‡ indicate that the samples were extracted from silica-gel dried leaves and herbarium specimens, respectively.

| Species | Voucher | No. reads | Plastome Length (bp) | Genbank accession | nrDNA Length (bp) | Genbank accession |
| --- | --- | --- | --- | --- | --- | --- |
| *Amelanchier ovalis* Moench | *F.R.Fosberg 35741* (US) | 9,726,571 | 159,695 | MK920297 | 6394 | MN215988 |
| *Amelanchier spicata* (Lam.) K.Koch | *J.Wen 12951* (US) | 6,385,440 | 159,965 | MK920292 | 6392 | MN215976 |
| *Aronia arbutifolia* (L.) Pers. | *J.Wen 13937* (US) | 7,814,435 | 159,777 | MN061996 | 6401 | MN215979 |
| *Chaenomeles cathayensis* (Hemsl.) C.K.Schneid. | *B.B.Liu 3909* (PE) | 6,612,128 | 159,907 | MN061997 | 6381 | MN215991 |
| *Cotoneaster submultiflorus* Popov | *PE Xizang Expedition 7046* (PE) | 6,216,071 | 159,822 | MK920286 | 6399 | MN215995 |
| *Cormus domestica* (L.) Spach | - | - | 133,216 | KY419956 | - | - |
| *Cotoneaster acuminatus* Lindl.^α^ | *PE-Xizang Expedition PE6623* (PE)† | 36,864,522 | 159,524 | MN577874 | 6335 | MN577908 |
| *Cotoneaster buxifolius* Wall. ex Lindl.^α^ | *B.B.Liu 2666* (PE)† | 35,154,686 | 159,563 | MN577892 | 6336 | MN577928 |
| *Cotoneaster franchetii* Bois | - | - | 133,130 | KY419994 | - | - |
| *Cotoneaster frigidus* Wall. ex Lindl.^α^ | *PE-Xizang Expedition 2965* (PE)† | 49,232,938 | 159,626 | MN577875 | 6331 | MN577909 |
| *Cotoneaster horizontalis* Decne.^α^ | *B.B.Liu 2059* (PE)† | 31,234,790 | 159,548 | MN577893 | 6333 | MN577929 |
| *Cotoneaster horizontalis* Decne. 1 | - | - | 133,181 | KY419917 | - | - |
| *Cotoneaster microphyllus* Wall. ex Lindl.^α^ | *PE-Xizang Expedition PE5890* (PE)† | 37,464,574 | 159,662 | MN577873 | 6332 | MN577907 |
| *Cotoneaster rubens* W.W.Sm.^α^ | *PE-Xizang Expedition PE6670* (PE)† | 29,695,566 | 159,525 | MN577895 | 6333 | MN577931 |
| *Cotoneaster salicifolius* Franch. | - | - | 133,050 | KY419943 | - | - |
| *Cotoneaster salicifolius* var. *henryanus* (C.K.Schneid.) T.T.Yu^α^ | *B.B.Liu 2241* (PE)† | 34,546,394 | 159,746 | MN577863 | 6334 | MN577896 |
| *Cotoneaster silvestrii* Pamp.^α^ | *B.B.Liu P1987-2* (PE)† | 30,721,508 | 159,796 | MN577894 | 6332 | MN577930 |
| *Cotoneaster submultiflorus* Popov | *PE Xizang Expedition 7046* (PE) | 6,216,071 | 159,822 | MK920286 | 6409 | MN215995 |
| *Cotoneaster taylorii* T.T.Yu^α^ | *Y.S.Chen et al. 13-1666* (PE)† | 25,966,312 | 159,607 | MN577872 | 6331 | MN577905 |
| *Crataegus marshallii* Eggl. | *J.B.Nelson 26961* (US) | 5,557,449 | 159,660 | MK920293 | 6401 | MN215977 |
| *Cydonia oblonga* Mill. | *B.B.Liu 3873* (PE) | 11,293,888 | 159,643 | MN061993 | 6210 | MN216014 |
| *Dichotomanthes tristaniicarpa* Kurz | *B.B.Liu & F.Zhao 3958* (PE) | 46,445,086 | 160,682 | MN577869 | 6333 | MN577902 |
| *Docynia delavayi* (Franch.) C.K.Schneid. | *B.B.Liu & F.Zhao 3959* (PE) | 11,288,423 | 159,823 | MN216025 | 6206 | MN216015 |
| *Eriobotrya bengalensis* var. *angustifolia* Cardot | - | - | 133,134 | KY419922 | - | - |
| *Eriobotrya cavaleriei* (H.Lév.) Rehder | *B.B.Liu 2585* (PE) | 8,957,501 | 159,210 | MK920283 | 6401 | MN215982 |
| *Eriobotrya deflexa* (Hemsl.) Nakai | *H.M.Li & B.B.Liu 201818* (PE) | 4,706,950 | 159,361 | MK920282 | 6402 | MN215978 |
| *Eriobotrya henryi* Nakai^α^ | *J.S.Yang 91-002* (PE)† | 32,006,000 | 159,631 | MN577880 | 6331 | MN577914 |
| *Eriobotrya japonica* (Thunb.) Lindl. 1 | - | - | 159,137 | KT633951 | - | - |
| *Eriobotrya japonica* (Thunb.) Lindl. 2 | - | - | 159,156 | KY085905 | - | - |
| *Eriobotrya japonica* (Thunb.) Lindl.^α^ | *H.M.Li & B.B.Liu 201819* (PE)† | 26,441,336 | 159,156 | MN577877 | 6330 | MN577911 |
| *Eriobotrya malipoensis* K.C.Kuan^α^ | *Beijing Exped. 893033* (PE)‡ | 37,980,270 | 156,994 | MN577881 | 6330 | MN577915 |
| *Eriobotrya obovata* W.W.Sm.^α^ | *W.Q.Yin 580* (PE)‡ | 44,659,146 | 157,229 | MN577882 | 6332 | MN577916 |
| *Eriobotrya salwinensis* Hand.-Mazz.^α^ | *Qingzang Exped. 7390* (PE)‡ | 32,467,320 | 159,488 | MN577883 | 6332 | MN577918 |
| *Eriobotrya seguinii* (H.Lév.) Cardot ex Guillaumin^α^ | *Z.S.Zhang & Y.T.Zhang 4328* (PE)‡ | 40,124,744 | 159,450 | MN577884 | 6330 | MN577919 |
| *Eriobotrya seguinii* (H.Lév.) Cardot ex Guillaumin 1^α^ | *Z.S.Zhang & Y.T.Zhang 3983* (PE)‡ | 28,106,486 | 159,459 | MN577885 | 6330 | MN577920 |
| *Eriolobus trilobatus* (Labill. ex Poir.) M.Roem. | - | - | 160,207 | KX499858 | - | - |
| *Gillenia trifoliata* (Pursh) DC.^β^ | *B.B.Liu 4677* (US)† | 9,828,138 | 159,400 | MN068252 | 6351 | MN577923 |
| *Hesperomeles glabrata* Kunth | *P.E.Berry 4561* (US) | 6,987,622 | 160,176 | MK920298 | 6398 | MN216003 |
| *Heteromeles arbutifolia* (Lindl.) M.Roem. 1 | - | - | 134,351 | KY419965 | - | - |
| *Heteromeles arbutifolia* (Lindl.) M.Roem. | *J.Wen 13512* (US) | 5,292,425 | 160,261 | MK920281 | 6395 | MN215975 |
| *Kageneckia oblonga* Ruiz & Pav.^β^ | *M.Mahu & C.H.Badilla* 10358 (US)‡ | 10,287,426 | 159,409 | MN068266 | 6340 | MN577932 |
| *Lindleya mespiloides* Kunth^β^ | *H. et al. s.n.* (US)‡ | 8,201,376 | 158,697 | MN068248 | 6337 | MN577906 |
| *Malacomeles denticulata* (Kunth) Decne. | - | - | 133,418 | KY419982 | - | - |
| *Malus baccata* (L.) Borkh. | *J.Wen 14050* (US) | 6,088,145 | 160,024 | MK896774 | 6395 | MN215980 |
| *Malus doumeri* (Bois) A.Chev.^α^ | *B.B.Liu & T.Wang 4665* (PE)† | 27,624,492 | 159,654 | MN577878 | 6329 | MN577912 |
| *Malus florentina* C.K.Schneid. | - | - | 159,672 | KX499856 | - | - |
| *Malus ioensis* (Alph.Wood) Britton | *W.H.Horr 3606* (US) | 7,906,450 | 159,841 | MN062004 | 6399 | MN215985 |
| *Malus sieversii* (Ledeb.) M.Roem. | *X.X.Zhou 218754* (PE) | 6,548,652 | 159,895 | MN061985 | 6394 | MN215992 |
| *Mespilus germanica* L. | *M.D.Tidestrom* 14120 (US) | 6,117,326 | 159,616 | MK920295 | 6405 | MN215986 |
| *Osteomeles schwerinae* C.K.Schneid. | *D.Y.Hong et al. 3429* (PE) | 5,995,892 | 159,930 | MN062000 | 6399 | MN215990 |
| *Peraphyllum ramosissimum* Nutt. | - | - | 133,838 | KY420011 | - | - |
| *Phippsiomeles mexicana* (Baill.) B.B.Liu & J.Wen | *R.Mevaugh 13607* (US) | 7,510,758 | 160,927 | MN062003 | 6408 | MN216002 |
| *Photinia beckii* C.K.Schneid.^α^ | *Anshun Expedition 1976* (PE)‡ | 41,865,748 | 159,230 | MN577889 | 6329 | MN577925 |
| *Photinia davidiana* (Decne.) Cardot | - | - | 133,848 | KY420003 | - | - |
| *Photinia glabra* (Thunb.) Maxim. | *B.B.Liu P1901-2* (PE) | 5,868,944 | 159,571 | MK920277 | 6208 | MN216020 |
| *Photinia integrifolia* Lindl.^α^ | *X.H. Jin et al. ST1585* (PE)† | 30,994,936 | 159,654 | MN577879 | 6330 | MN577913 |
| *Photinia lanuginosa* T.T.Yu^α^ | *Z.C.Luo 730* (PE)‡ | 37,926,324 | 160,184 | MN577890 | 6330 | MN577926 |
| *Photinia lochengensis* T.T.Yu^α^ | *G.R.Long 89009* (PE)‡ | 30,171,458 | 160,201 | MN577888 | 6333 | MN577924 |
| *Photinia prionophylla* (Franch.) C.K.Schneid.^α^ | *Z.D.Fang et al. 20-427* (PE)‡ | 39,155,912 | 160,333 | MN577891 | 6330 | MN577927 |
| *Photinia prunifolia* (Hook. & Arn.) Lindl. | *X.X.Zhou 1164* (PE) | 7,309,854 | 159,757 | MK920279 | 6210 | MN216022 |
| *Photinia serratifolia* (Desf.) Kalkman^α^ | *B.B.Liu 2408* (PE)† | 39,261,848 | 160,254 | MN577865 | 6331 | MN577898 |
| *Photinia taishunensis* G.H.Xia, L.H.Lou & S.H.Jin | *B.B.Liu P1950-5* (PE) | 6,088,121 | 159,572 | MK920278 | 6208 | MN216021 |
| *Pourthiaea villosa* (Thunb.) Decne. | *B.B.Liu P1919-3* (PE) | 12,844,729 | 160,401 | MN061989 | 6207 | MN216009 |
| *Pseudocydonia sinensis* (Thouin) C.K.Schneid.^α^ | *B.B.Liu 3981* (PE)† | 37,338,914 | 159,445 | MN577871 | 6340 | MN577904 |
| *Pyracantha fortuneana* (Maxim.) H.L.Li | *D.Y.Hong et al. 3443* (PE) | 11,749,026 | 160,388 | MK920290 | 6217 | MN216013 |
| *Pyrus communis* L.^α^ | *FLPH Sichuan Expedition 151081* (PE)† | 49,981,000 | 160,171 | MN577870 | 6330 | MN577903 |
| *Rhaphiolepis ferruginea* F.P.Metcalf^α^ | *B.B.Liu P1900-3* (PE)† | 48,628,646 | 159,515 | MN577866 | 6330 | MN577899 |
| *Rhaphiolepis impressivena* Masam.^α^ | *T.C.Chen 11873* (PE)‡ | 26,218,960 | 159,554 | MN577886 | 6330 | MN577921 |
| *Rhaphiolepis indica* (L.) Lindl. ex Ker Gawl. 1 | - | - | 133,026 | KY419927 | - | - |
| *Rhaphiolepis indica* (L.) Lindl. ex Ker Gawl.^α^ | *B.B.Liu 2592* (PE)† | 31,391,422 | 159,466 | MN577864 | 6330 | MN577897 |
| *Rhaphiolepis lanceolata* H.H.Hu^α^ | *B.B.Liu 2153* (PE)† | 29,048,960 | 159,688 | MN577867 | 6330 | MN577900 |
| *Rhaphiolepis major* Cardot^α^ | *Wuyi Exped. 2188* (PE)‡ | 36,411,688 | 159,528 | MN577887 | 6330 | MN577922 |
| *Rhaphiolepis salicifolia* Lindl.^α^ | *H.M.Li & B.B.Liu 201815* (PE)† | 28,516,636 | 159,552 | MN577876 | 6330 | MN577910 |
| *Rhaphiolepis umbellata* (Thunb.) Makino 1 | - | - | 133,239 | KY419931 | - | - |
| *Rhaphiolepis umbellata* (Thunb.) Makino^α^ | *B.B.Liu 1951* (PE)† | 49,010,820 | 159,547 | MN577868 | 6330 | MN577901 |
| *Sorbus aria* (L.) Crantz | *H.B.Urdet s.n.* (US) | 9,358,325 | 160,222 | MN061998 | 6393 | MN216005 |
| *Sorbus chamaemespilus* (L.) Crantz | *A.Chrtkova-Zertova* et al. s.n. (US) | 9,775,901 | 160,326 | MN061999 | 6393 | MN216006 |
| *Sorbus commixta* Hedl. | *M.Li HG019* (CDBI) | 5,845,940 | 159,952 | MK920288 | 6400 | MN215997 |
| *Stranvaesia bodinieri* (H.Lév.) B.B.Liu & J.Wen | *B.B.Liu P1941-3* (PE) | 8,412,063 | 159,865 | MK920276 | 6212 | MN216019 |
| *Stranvaesia nussia* (Buch.-Ham. ex D.Don) Decne. | *D.H.Nicolson 2719* (US) | 9,944,353 | 159,937 | MK920284 | 6399 | MN215989 |
| *Stranvaesia oblanceolata* (Rehder & E.H.Wilson) Stapf | *780Gongcheng 873* (PE) | 10,625,039 | 159,808 | MK920280 | 6212 | MN216023 |
| *Torminalis clusii* M.Roem. | *E.Herbier s.n.* (CDBI) | 5,969,532 | 160,379 | MN062005 | 6472 | MN215999 |
| *Vauquelinia australis* Standl.^β^ | *W.Hess & G.Wilhelm s.n.* (US)‡ | 10,443,976 | 159,813 | MN068250 | 6337 | MN577917 |
